# Downregulation of NKG2DLs by TGF-β in human lung cancer cells

**DOI:** 10.1186/s12865-021-00434-8

**Published:** 2021-07-12

**Authors:** Young Shin Lee, Hojung Choi, Hae-Ryung Cho, Woo-Chang Son, You-Soo Park, Chi-Dug Kang, Jaeho Bae

**Affiliations:** 1 Department of Biochemistry, Pusan National University School of Medicine, Yangsan, 50162 South Korea; 2 PNU BK21 Plus Biomedical Science Education Center, Pusan National University School of Medicine, Yangsan, 50612 South Korea; 3 Department of Research Center, Dongnam Institute of Radiological and Medical Sciences, Gijang, Busan, 46033 South Korea

**Keywords:** NKG2D ligands, Transforming growth factor beta, Matrix metalloproteinase

## Abstract

**Background:**

Transforming growth factor beta (TGF-β) is a typical immuno-inhibitory cytokine and highly secreted by lung cancer cells. It was supposed that its immunosuppressive effects to NK cell might be related with the altered expression of activating and inhibitory molecules in lung cancer cells. In this study, we examined the expression of NKG2DLs, PD-L1 and PD-L2 in lung cancer cells after treatment of TGF-β and a TGF-β inhibitor, Galunisertib (LY2157299).

**Results:**

TGF-β reduced the level of surface proteins of five NKG2DLs without altered transcription levels in lung cancer cells. Galunisertib reversed the effect of TGF-β on the expression of NKG2DLs. Since MMP inhibitors, MMPi III and MMP2 inhibitor I, restored the reduced expression of NKG2DLs after treatment of TGF-β, it was thought that TGF-β induced the expression of MMP2 which facilitated the shedding of the NKG2DLs in cancer cells. However, the expression of PD-L1, L2 were not changed by treatment with TGF-β or Galunisertib.

**Conclusions:**

Therefore, inhibition of TGF-β might reverse the immunosuppressive status on immune cells and restore NK cell mediated anticancer immune responses by upregulation of NKG2DLs in cancer cells.

**Supplementary Information:**

The online version contains supplementary material available at 10.1186/s12865-021-00434-8.

## Background

Lung cancer is one of the most commonly diagnosed cancer and also the leading cause of cancer-associated mortality [1]. Even with more advanced chemotherapeutic agents and molecularly targeted drugs, the prognosis of this disease is still poor due to limited treatment efficiency [2, 3]. Thus, given the higher recurrence and mortality rates, novel therapeutic strategies are warranted in order to improve the outcome of patients with lung cancer.

Natural-killer group 2, member D (NKG2D), is expressed by human NK cells and some kinds of T cells, and transduces activating signals through binding to the NKG2DLs [4]. In this process, upregulation of NKG2DLs could activate the NK cells and evoke immune responses against the target cells [5]. Programmed cell death protein 1 (PD-1) is an immune checkpoint molecule and transduces inhibitory signaling which is expressed by mainly lymphocytes [6]. It binds to PD-L1 and PD-L2 on target cells, and decrease anti-cancer immune responses [7]. Since the activity of NK cells were modulated by the signaling balance derived from inhibitory and activating receptors, it was suggested that these NKG2DLs and PD-L1/2 might significantly influence on the killing ability of NK cells against cancer cells.

Among various immunomodulatory factors, transforming growth factor-β (TGF-β) is a potent cytokine with immune suppressive effects including the negative regulation of lymphocyte proliferation, differentiation and survival [8] and TGF-β inhibitor may attenuated the immune suppressive effects [9–11].. It was known that TGF-β could inhibit the activity of natural killer (NK) cells and susceptibility of cancer cells to NK cells [12, 13]. In addition, TGF-β regulates chemotaxis and the activity of other immune cells such as dendritic cells, macrophages, mast cells and granulocytes [8]. Therefore, TGF-β is associated with tumor growth and malignant progression in various types of cancers [14–16]. It was known that promoted metastasis and invasion of cancer cells through TGF-β signaling was associated with the increased expression and activity of matrix metalloproteinases (MMPs) [17, 18]. MMPs are zinc-dependent enzymes which play an important role in extracellular matrix degradation in the tumor microenvironment [19]. In addition, some kinds of metalloproteinase family facilitated the shedding and reduction of surface expression of NKG2DLs [20, 21]. Since TGF-β was highly secreted in lung cancer cells [22], it was supposed that TGF-β might change the expression of signaling on NK cells through the altered expression of NKG2DLs and PD-L1/L2. Therefore, high expression of MMPs might suppress NK cell-mediated anti-cancer immune responses.

In this study, it was evaluated that TGF-β and TGF-β inhibitor could altered expression of NKG2DLs and PD-L1/L2. In addition, a possible modulating molecule, MMP2 could mediate the expression of NKGD2DLs through TGF-β signaling. Finally, it was investigated that TGF-β inhibitor could enhance the susceptibility of lung cancer cells to NK cell.

## Results

### TGF-β decreased the surface expression of NKG2DLs in lung cancer cells

**Fig. 1:**
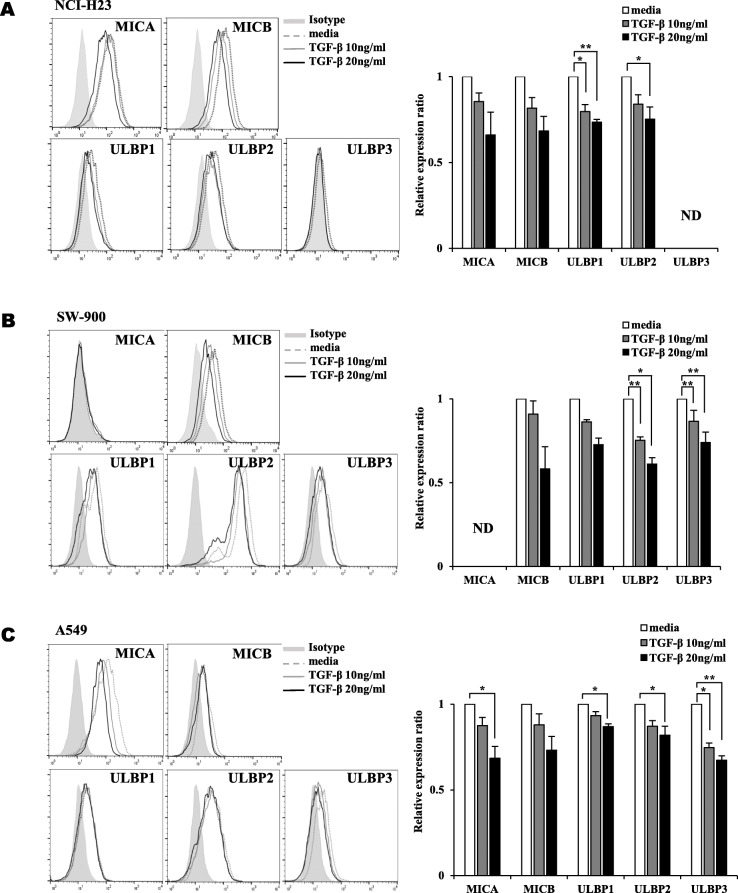
Decrease surface expression levels of some kinds of NKG2DLs by treatment with TGF-β. The surface expression levels of NKG2DLs were showed as histograms and graphs in **(A)** NCI-H23, **(B)** SW-900 and **(C)** A549 lung cancer cells, which were treated with TGF-β (10 ng/ml, 20 ng/ml) for 24 h. A representative data was shown. The relative expression ratios were calculated from mean fluorescence intensities (MFI) of treated cells compared to those of control cells. Filled gray, dotted gray, gray and black lines represent isotype, media control, TGF-β 10 ng/ml treatment and TGF-β 20 ng/ml treatment, respectively. The experiments were performed three times. (*p* < 0.05, *; *p* < 0.01, **)

The surface expression of NKG2DLs in lung cancer cells was analyzed using flow cytometry after treatment with TGF-β. The surface expression levels of five NKG2DLs including ULBP1 and ULBP2 were decreased by - 0.3-fold after treatment with 20 ng/ml TGF-β. ULBP1 was supressed at 10 ng/ml TGF- β in NCI-H23 cells (Fig. 1A). The surface expression levels of ULBP1, ULBP2 and ULBP3 were decreased by 0.3-fold, 0.4-fold and 0.3-fold, respectively, after treatment with 10 ng/ml TGF-β in SW-900 cells. The reduction of these three kinds of NKG2DLs was more profound. (Fig. 1B). MICA and ULBP1/2/3 were decreased by 0.3-fold, 0.1-fold, 0.2-fold and 0.3-fold, respectively, after treatment with 20 ng/ml TGF-β. ULBP3 was supressed at 10 ng/ml TGF- β in A549 cells (Fig. 1C). ULBP3in NCI-H23 cells and MICA in SW-900 cells were not detected and marked by ND. These results suggest that TGF-β could affect the surface expression of NKG2DLs in lung cancer cells. The kinds of NKG2DLs which were decreased after treatment with TGF-β were different depending on the cell lines.

### Galunisertib reversed the surface expression of NKG2DLs in lung cancer cells

**Fig. 2:**
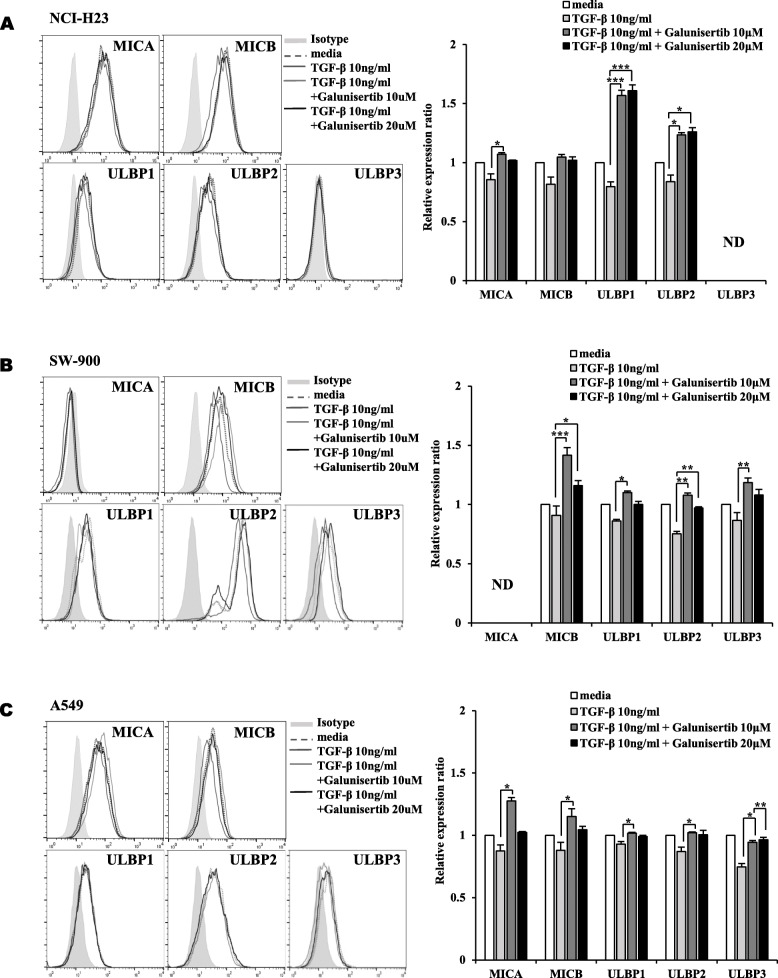
Restored surface expression levels of NKG2DLs by blocking of TGF-β signaling. The surface expression levels of NKG2DLs were analyzed using flow cytometry in **(A)** NCI-H23, **(B)** SW-900 and **(C)** A549 lung cancer cells. The lung cancer cells were treated with TGF-β (10 ng/ml) and followed by Galunisertib (10 μM, 20 μM) for 24 h. The relative expression ratios were calculated from mean fluorescence intensities (MFI) of treated cells compared to those of control cells. Filled gray, dotted gray, dark gray, light gray and black lines represent isotype, media control, TGF-β 10 ng/ml treatment, TGF-β 10 ng/ml with Galunisertib 10 μM treatment and TGF-β 10 ng/ml with Galunisertib 20 μM treatment, respectively. The experiments were performed three times. (*p* < 0.05, *; *p* < 0.01, **; *p* < 0.001, ***)

To block the role of TGF-β, three kinds of lung cancer cells were treated with a TGF-β inhibitor, Galunisertib, and the surface expression of five NKG2DLs was analyzed by flow cytometry. Galunisertib reversed the effects of TGF-b in surface expression of NKG2DLs in lung cancer cells. In NCI-H23 cells, the surface expression of MICA, ULBP1 and ULBP2 was increased by TGF-β inhibitor (Fig. 2A). In SW-900 cells, the surface expression of MICB and ULBP1/2/3 was increased by TGF-β inhibitor (Fig. 2B). In A549 cells, five kinds of NKG2DLs were reversed by TGF-β inhibitor (Fig. 2C). As results from Figs. 1 and 2, it was supposed that TGF-β signaling might regulate the expression of NKG2D ligands in lung cancer cells.

### Combined treatment of TGF-β and Galunisertib did not alter the transcription levels of NKG2DLs in lung cancer cells

**Fig. 3:**
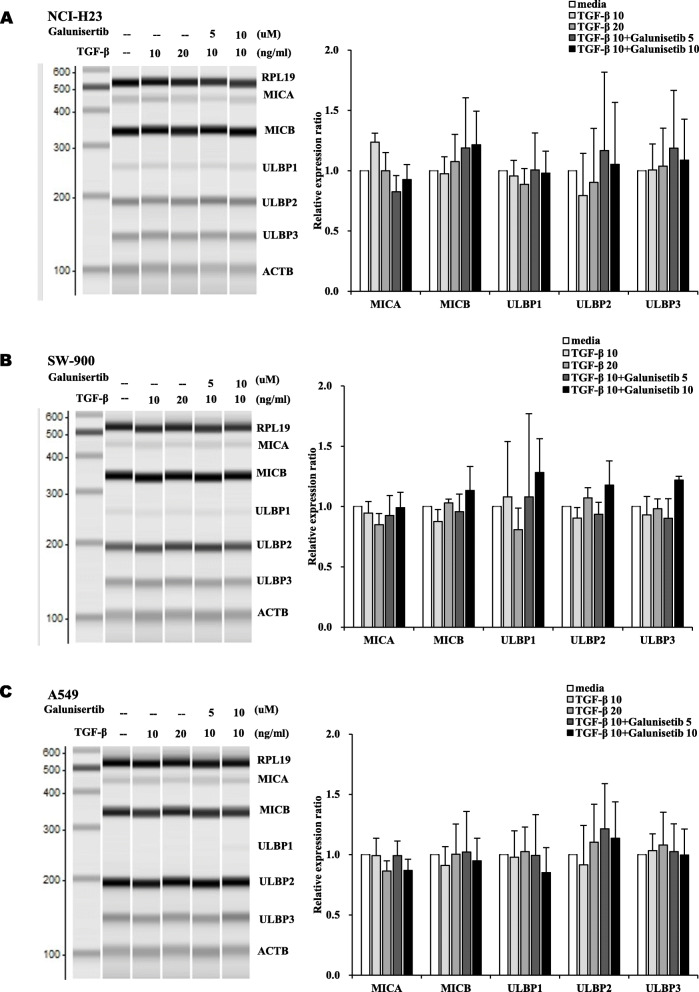
No significant the changes of transcription of NKG2DLs by combined treatment of TGF-β and Galunisertib in lung cancer cells. The transcription levels of NKG2DLs were analyzed in **(A)** NCI-H23, **(B)** SW-900 and **(C)** A549 lung cancer cells by multiplex RT-PCR. The flung cancer cells were treated with TGF-β (10 and 20 ng/ml) and Galunisertib (5 and 10 μM) for 18 h. The PCR products were separated and quantified by MultiNA. All experiments were done in triplicate. Changes of transcriptions were normalized by ACTB and presented as the mean fold changes compared to media treated groups. The experiments were performed three times

The transcription levels of NKG2DLs were analyzed by multiplex RT-PCR. When treated with TGF-β and Galunisertib, the transcription levels of NKG2DLs in three lung cancer cells were not changed significantly (Fig. 3). Therefore, TGF-β signaling may modulate the expression of NKG2DLs at post-transcriptional level without significant changes of transcription.

### Treatment with TGF-β and Galunisertib altered the expression of MMP2

Since previous results showed that TGF-β regulates the surface expression of NKG2D s, it was needed to investigate how TGF-β regulates the expression of NKG2DLs. It was known that many kinds of MMPs were induced by TGF-β and some kinds of MMPs such as MMP2, MMP9 and MMP14 could reduce the surface expression of NKG2DLs [19, 21, 23, 24]. Therefore, it was supposed that the surface expression of NKG2DLs might be regulated by the role MMPs induced by TGF-β. In these studies, we investigated the changes of MMPs through TGF-β signaling which were related with the reduction of surface NKG2DLs.

**Fig. 4:**
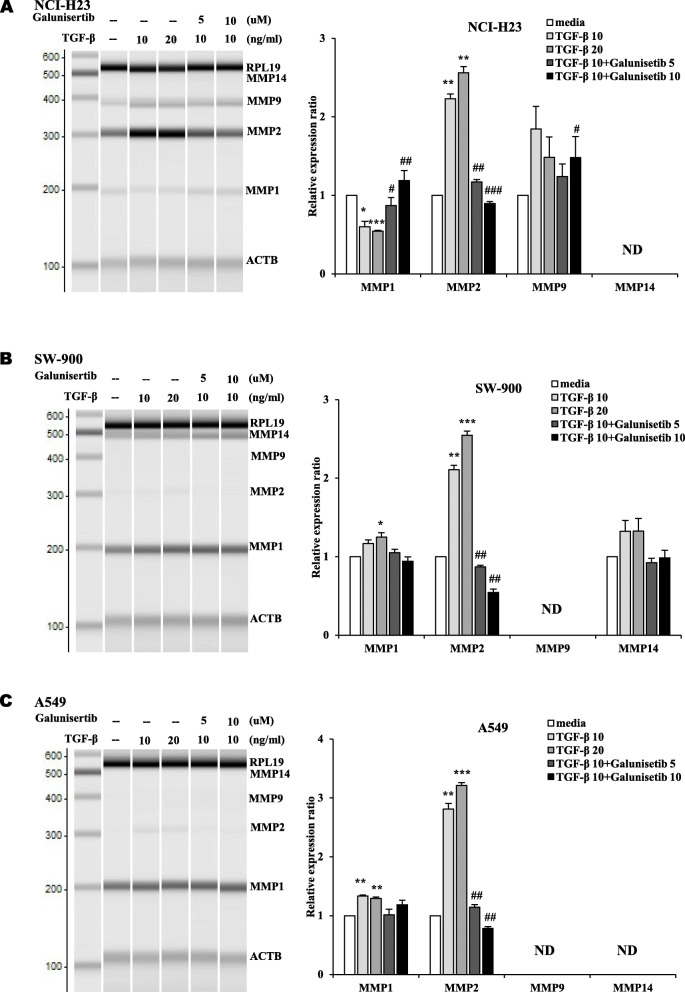
The transcriptions of MMPs were regulated by treatment with TGF-β and Galunisertib. The transcription levels of MMPs were analyzed in **(A)** NCI-H23, **(B)** SW-900 and **(C)** A549 lung cancer cells by multiplex RT-PCR. Lung cancer cells were treated with TGF-β (10 and 20 ng/ml) and Galunisertib (5 and 10 μM) for 18 h. The PCR products were separated and quantified by MultiNA. All experiments were done in triplicate. The changes of transcripts were compared to media treated groups after treatment with TGF-β without Galunisertib. The changes of transcription levels were compared to TGF-β treated groups after combined treatment with TGF-β with Galunisertib. The experiments were performed three times. (*# P < 0.05, **## *P* < 0.01, ***### *P* < 0.001)

**Fig. 5:**
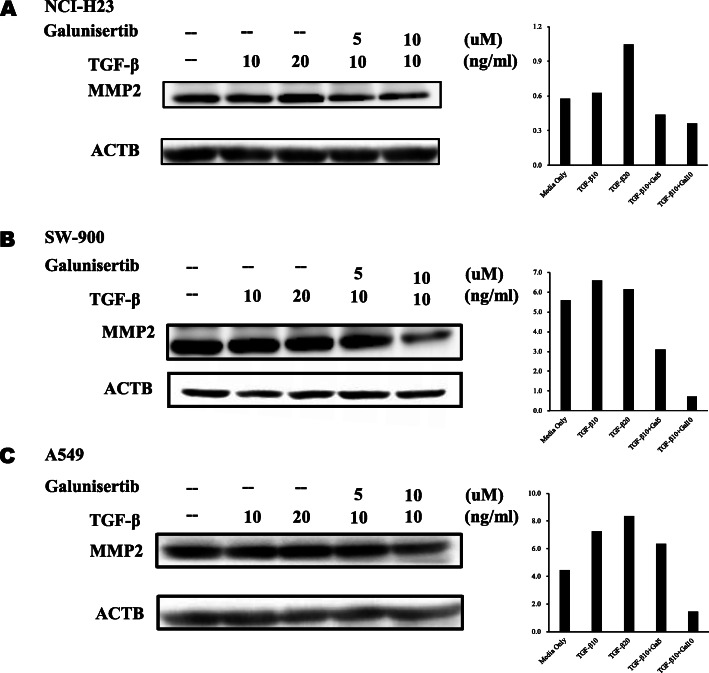
The level of MMP2 was modulated by treatment with TGF-β and Galunisertib. The MMP2 at protein level were analyzed in **(A)** NCI-H23, **(B)** SW-900 and **(C)** A549 lung cancer cells by western blot. Lung cancer cells were treated with TGF-β (10 and 20 ng/ml) and Galunisertib (5 and 10 μM) for 18 h. Bands were cropped from original images (Fig. S4) and intensity was measured using the ImageJ software and the expression levels were calculated by ratio against β-actin. The experiments were performed three times and showed representative data

The transcription of MMP2 was significantly increased by treatment with TGF-β in three lung cancer cells (Figs. 4 and 5). The other MMPs including MMP1, MMP9 and MMP14 were differentially changed in three lung cancer cells depending on specific cell types. MMP1 was increased in SW-900 cells and A549 cells by treatment with TGF-β 10 ng/ml and 20 ng/ml (Fig. 4B and C). MMP9 in NCI-H23 cells, and MMP1 in SW-900 cells were also increased by treatment with TGF-β (Fig. 4A and B). When TGF-β and Galunisertib were cotreated, increased MMPs were reversed except MMP1 in SW-900 and A549. It was demonstrated that TGF-β induced the expression of MMP2 and Galunisertib reversed the changes of MMPs in three kinds of lung cancer cells (Fig. 4). Interestingly, the expression of MMP1 in NCI-H23 cells was decreased by treatment with TGF-β (Fig. 4A). MMP14 in NCI-H23 cells, MMP9 in SW-900 cells, and MMP9 and MMP14 in A549 cells were not detected and marked as ND.

When the protein levels of MMP2 were measured, TGF-β induced the expression of MMP2 and Galunisertib reversed its level at three kinds of lung cancer cells (Fig. 5). It was supposed that high level of MMP2 might reduce the surface NKG2DLs.

### MMP inhibitors increased the surface expression of NKG2DLs in lung cancer cells

**Fig. 6:**
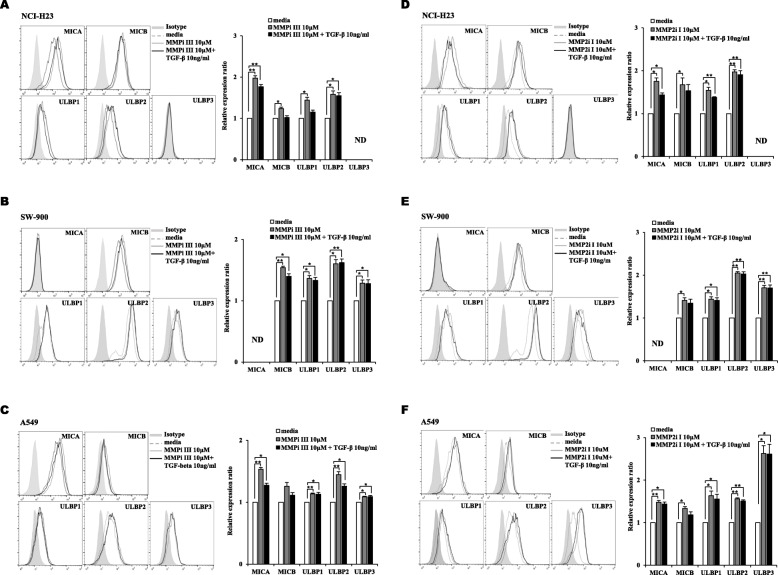
The induction of NKG2DLs by treatment with MMP inhibitors and limited induction by co-treatment with TGF-β in lung cancer cells. Surface expression levels of NKG2DLs were analyzed using flow cytometry in **(A, D)** NCI-H23, **(B, E)** SW-900 and **(C, F)** A549 lung cancer cells. Lung cancer cells were treated with MMP inhibitors and TGF-β (10 ng/ml) for 18 h. The relative expression ratios were calculated from mean fluorescence intensities (MFI) of treated cells compared to those of control cells. Filled gray, dotted gray, gray and black lines represent isotype, media control, MMP inhibitors 10 μM treatment and TGF-β 10 ng/ml with MMP inhibitors 10 μM treatment, respectively. The experiments were performed three times. (*p* < 0.05, *; *p* < 0.01, **)

To investigated the role of MMPs through TGF-β signaling, three lung cancer cells treated with MMP inhibitor III (MMPi III), and MMP2 inhibitor I (MMP2i I) which are a broad spectrum MMP inhibitor and MMP2 specific inhibitor, respectively. Both MMP inhibitors dramatically increased NKG2DLs except ULBP3 in NCI-H23 cells and MICA in SW-900 which was not detected any condition (Fig. 6). When MMP inhibitors were co-treated with TGF-β, the suppression of NKG2DLs was blocked partially in three lung cancer cell lines. Although it was treated with MMP2 inhibitor, the expression of NKG2DLs showed a tendency to decrease with treatment of TGF-β. It means that TGF-β has another mechanism to reduce the expression of NKG2DLs besides the induction of MMP2. In previous studies, it can be suggested that there are two possible mechanisms which are upregulation of proteasomal activity and induction of miRNA [25–27].

### Galunisertib reversed susceptibility of lung cancer cells to NK cells

**Fig. 7:**
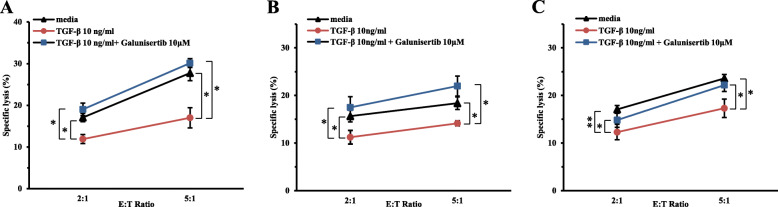
The enhanced NK cell-mediated cytotoxicity by treatment of Galunisertib. The susceptibility of lung cancer cells to NK-92 cells were analyzed using flow cytometry in **(A)** NCI-H23, **(B)** SW-900 and **(C)** A549 lung cancer cells. Lung cancer cells were treated with TGF-β (10 ng/ml) and Galunisertib (10 μM) for 24 h. Black, red and blue lines represent media control, TGF-β treatment and TGF-β with Galunisertib treatment, respectively. The experiments were performed three times. (*p <* 0.05, *; *p <* 0.01, **)

Since TGF-β downregulated the expression of NKG2DLs in lung cancer cells, it might repress the susceptibility of lung cancer cells to NK-92 cells. By treatment of TGF-beta, NK cell-mediated cytotoxicity was suppressed. The susceptibility of lung cancer cells to NK-92 cells was reversed by co-treatment of Galunisertib in three lung cancer cells (Fig. 7).

## Discussion

TGF-β signaling plays a crucial role in various tumor microenvironments and performs a variety of functions such as cell proliferation, differentiation, apoptosis, extracellular matrix reorganization and epithelial-mesenchymal transition (EMT) [28]. In addition, TGF-β is a cytokine which has immune suppressive effects [8]. In previous studies, TGF-β directly suppressed cytotoxic T lymphocyte and induced regulatory T cells. TGF-β also inhibited NK cell and B cell proliferation and functions [29–33]. Therefore, it is thought that the inhibition of TGF-β is important to restore function of NK cells and suppress the cancer progression.

NKG2DLs in cancer cells are known to promote immune responses by binding to NKG2D in immune effector cells such as some kinds of T cells and NK cells [5, 34]. Although it was known that TGF-β could inhibit the activity of NK cells and the susceptibility of cancer cells to NK cells [35], the mechanism by which TGF-β affect the expression of NKG2DLs in lung cancer cells has not been identified. We supposed that TGF-β might affect the expression of activating ligands such as NKG2DLs and inhibitory ligands such as PD-L1/L2 in lung cancer cells. In this study, it was investigated whether NKG2DLs and PD-L1/L2 in lung cancer cells could be regulated by TGF-β signaling. As a result, TGF-β and a TGF-β inhibitor, Galunisertib, could regulate the surface expression of NKG2DLs in lung cancer cells. The transcription levels of NKG2DLs were not related with TGF-β signaling. Although the exact mechanism of TGF-β in regulation of NKG2DLs is not clear, it was thought that the main regulation step is post-transcriptional level.

There are many reports that TGF-β stimulates the expression of variety of MMPs in mammary carcinoma, squamous cell carcinoma (SSC), melanoma and other types of cancer cells [17, 36–38]. Since inhibition of MMPs could increase the expression levels of NKG2DLs [19, 38, 39], it was suggested that MMPs mediated the regulation of NKG2DLs through TGF-β signaling.

In this study, it was demonstrated that the mRNA and protein levels of MMP2 were increased by treatment with TGF-β and reversed by co-treatment with Galunisertib. The inhibition of MMPs by treatment with MMPi III and MMP2i I upregulated the surface expression of NKG2DLs which were suppressed by TGF-β. These results suggest that MMP2 might be a main regulator in the expression of NKG2DLs through TGF-β signaling. TGF-β induced the expression of MMP2 commonly in three lung cancer cells and other MMPs were regulated differentially depends on cell types.

It has been suggested that metalloproteinase family including MMPs and ADAMs could increase the level of soluble NKG2DLs and decrease the level of surface NKG2DLs simultaneously [20, 21]. It was also reported that MMP2 in renal carcinoma cells, MMP9 and MMP14 in osteosarcoma cells could increase soluble MICA [23, 24, 40, 41] and MMP9 specific inhibitor could increase MICA/B and ULBP2 in some kinds of gastric cancer cells [19].

NK cells are cytotoxic against many cancer cells and the cytotoxicity of NK cells is regulated by the signal transduction balance between activation and inhibition receptors [34, 42–45]. Thus, beside of the induction of activating ligands such as NKG2DLs in cancer cells, the reduction of inhibition ligands is necessary to enhanced NK cell mediated anticancer responses. It was investigated whether a kind of inhibition ligands, PD-L1 and PD-L2, in cancer cells could be regulated by TGF-β signaling. In result, TGF-β signaling did not influence on the expression of PD-L1 and PD-L2 (Fig. S2).

Many kinds of cancer overexpress TGF-β and high levels of TGF-β in cancer patients frequently associated with poor prognosis. Recently anti TGF-β therapy has been tried to control cancer progression. We supposed that anti TGF-β therapy might inhibit cancer progression as well as restore anti-cancer immune responses through reactivation of immune cells and enhance the susceptibility of cancer cells.

## Conclusion

Stimulation of TGF-β signaling could decrease the expression of NKG2DLs in lung cancer cells which might be related with increased expression of MMP2. Therefore, inhibition of TGF-β might block the immunosuppressive effects on immune cells and restore NK cell mediated anticancer immune responses by upregulation of NKG2DLs in cancer cells.

## Methods

### Cell lines and reagents

Three human lung cancer cell lines were used in this study including A549, NCI-H23, and SW-900. These cell lines were obtained from the Korean Cell Line Bank (Seoul, South Korea) and were maintained in RPMI-1640 medium supplemented with 10% fetal bovine serum (Gibco, Grand Island, NY, USA), 2 mM L-glutamine, 100 mg/mL streptomycin, and 100 U/mL penicillin. All cell lines were cultured at 37 °C in a humidified atmosphere containing 5% CO_2_ [46].

Recombinant human TGF-β was purchased from R&D systems, Inc. (Minneapolise, MN, USA), dissolved at 20 μg/ml in sterile 4 mM HCl containing 1 mg/ml bovine serum albumin and used at 10 and 20 ng/ml doses. Galunisertib were purchased from Selleckchem (Houston, TX, USA), dissolved at 10 mM in dimethyl sulfoxide and used at 5, 10, and 20 μM doses. MMP Inhibitor III, a pan inhibitor of MMPs, and MMP2 inhibitor I, a specific inhibitor of MMP2, were purchased from Calbiochem (Merck KGaA, Darmstadt, Germany) and dissolved at 10 mM in dimethyl sulfoxide and used at 10 μM dose. \.

### Total RNA extraction and multiplex reverse transcription (RT)-PCR

Total RNA extraction and qPCR were performed as described by previous methods [47]. Briefly, total RNA was extracted from the cells using the RNeasy® Mini kit (Qiagen, Hilden, Germany), according to the manufacturer’s instructions. cDNA was synthesized from 1 μg extracted total RNA, using 100 pmol random primers (Takara Bio Inc., Otsu, Japan) and 100 U M-MLV reverse transcriptase (Promega Corporation, Madison, WI, USA). The resulting cDNA was used in the PCR reaction, which was performed using the QIAGEN Multiplex PCR kit (Qiagen). Numerous primer pairs were used to investigate the mRNA expression levels of NKG2DLs [47]: MHC class-I polypeptide-related chain proteins MICA and MICB, UL-16 binding proteins (ULBP)1–3, MMPS: of matrix metalloproteinases (MMP)1, 2, 9 and 14; PD-1 ligands: Programmed death-ligand 1 (PD-L1), Programmed cell death 1 ligand 2 (PD-L2) (Bioneer Corporation, Daejeon, South Korea). β-actin and ribosomal protein L19 were used as the loading control and degradation marker, respectively. The PCR products were separated and quantified by MultiNA (Shimadzu, Tokyo, Japan).

### Flow cytometry

To determine the surface NKG2DLs on cancer cells, the cells were incubated with mouse anti-MICA, anti-MICB, anti-ULBP1–3 (R&D systems, Minneapolis, MN, USA), which were NKG2D ligand-specific monoclonal antibodies (mAbs) or the corresponding isotype controls at 1 μg/100 μl followed by incubation with PE goat anti-mouse Ig (BD Phamingen Inc., San Diego, CA, USA). The analysis was performed on the BD FACSCANTO II (Becton Dickinson and Company, Franklin Lakes, NJ, USA) using FlowJo software (BD Biosciences, Franklin Lakes, NJ, USA) and the cell surface expression was quantified by the value of the mean fluorescence intensities (MFI) obtained with the specific mAbs [48].

### Western blotting analysis

Western blotting analysis was performed to evaluate the expression of MMP. The cells were washed twice with cold phosphate buffered saline and lysed. Equal amounts of cell extracts were resolved by 4–20% SDS-PAGE and analyzed by Western blot. The separated proteins were transferred onto nitrocellulose membranes (Hybond-ECL, GE healthcare, Buckinghamshire, UK). In the next step, the membranes were blocked with 4% nonfat milk in Tris buffered saline containing 0.1% Tween 20 at room temperature. Proteins of interest were then detected with the primary antibodies (MMP 1 and 2: Cell signaling, Beverly, MA; MMP9: Santa Cruz Biotechnology, Santa Cruz, CA, USA) and with horseradish peroxidase (HRP)-conjugated secondary antibodies (Cell Signaling Technology, MA, USA) using an enhanced chemiluminescence detection system (Amersham™ ECL™ Select Western Blotting Detection Reagent; GE Healthcare) in accordance with the manufacturer’s instructions. Each blot was probed with an anti-β-actin antibody (Sigma-Aldrich, St. Louis, MO, USA). Bands were detected using AI 680 (Amersham™ Imager 680 – Blot and Gel Imager, GE Healthcare) and intensity quantification was performed using the ImageJ software (version 1.52; National Institutes of Health, Bethesda, MD, USA). The protein expressions in the treated cells were divided by the level of β-actin to calculate relative protein expression ratios.

### NK cell-mediated cytotoxicity

NK cell mediated cytotoxicity was determined using FC500 flow cytometer (Beckman Coulter, CA, USA). The lung cancer cells were stained with 50 mM carboxyfluorescein succinimidyl ester (CFSE) for 30 min at 37 °C and washed three times. NK 92 cells and CFSE stained lung cancer cells were co cultured for 4 h. Propidium iodide (PI) was added to the co cultured samples for identification of the dead cells. Cytotoxicity were calculated by formula of (CFSE+PI+ cells / CFSE+ cells) X 100 (%).

### Statistical analysis

To evaluate the altered gene expression levels, the mean folds of gene expressions among the groups and the standard error of the mean were calculated. For comparisons of the groups, a paired Student’s t-test was performed. The analysis was performed using the SPSS statistical package (version 14.0; SPSS Inc., IL, USA). *P* < 0.05 indicates a statistically significant difference in all of the experiments.

## Supplementary Information

**Additional file 1: Fig. S1** The direct effect of Galunisertib to the expression of NKG2D ligands on lung cancer cells; **A** NCI-H23, **B** SW-900, and **C** A549. **Fig. S2** The expression of PD-L1/2 on lung cancer cells; **A** NCI-H23, **B** SW-900, and **C** A549. **Fig. S3** The expression of ADAM10 and ADAM17 after TGF-β and Galunisertib treatment. **A** NCI-H23 cells; **B** SW-900 cells; **C** A549 cells. **FigureS4** The modulation of MMP2 by TGF-β and Galunisertib treatment. **A** MMP2 and actin in NCI-H23 cells; **B** MMP2 and actin in SW-900 cells; **C** MMP2 and actin in A549 cells.

## Data Availability

The datasets used and/or analyzed during the current study are available from the corresponding author on reasonable request.

## Abbreviations

EMT: Epithelial-mesenchymal transition
MFI: Mean fluorescence intensities
MIC: MHC class 1 chain-related protein
MMP: Matrix metalloproteinase
ND: Not detected
NKG2D: Natural-killer group2, member D
PD-L1/2: Programmed death ligand 1/2
TGF-β: Transforming growth factor beta
ULBP: UL16 binding protein
