# Supplementary material for: Downregulation of NKG2DLs by TGF-β in human lung cancer cells
Source: BMC Immunol. 2021 Jul 12;22:44. doi: 10.1186/s12865-021-00434-8 (PMC8273967; doi:10.1186/s12865-021-00434-8)
Supplement: Supplementary file 1 — Additional file 1: Fig. S1 The direct effect of Galunisertib to the expression of NKG2D ligands on lung cancer cells; A NCI-H23, B SW-900, and C A549. Fig. S2 The expression of PD-L1/2 on lung cancer cells; A NCI-H23, B SW-900, and C A549. Fig. S3 The expression of ADAM10 and ADAM17 after TGF-β and Galunisertib treatment. A NCI-H23 cells; B SW-900 cells; C A549 cells. FigureS4 The modulation of MMP2 by TGF-β and Galunisertib treatment. A MMP2 and actin in NCI-H23 cells; B MMP2 and actin in SW-900 cells; C MMP2 and actin in A549 cells. [file 12865_2021_434_MOESM1_ESM.pdf]

Downregulation of NKG2DLs by TGF-β in Human Lung Cancer Cells

Young Shin Lee[a#], Hojung Choi[a#], Hae-Ryung Cho[a,b], Woo-Chang Son[c], Yu-Soo Park[c], Chi-Dug Kang[a], Jaeho Bae[a,b*]

[a]Department of Biochemistry, Pusan National University School of Medicine, Yangsan, 50162, South Korea.
[b]PNU BK21 Plus Biomedical Science Education Center, Pusan National University School of Medicine, Yangsan, 50612, Korea.
[c]Department of Research Center, Dongnam Institute of Radiological and Medical Sciences, Gijang, Busan, 46033, Korea

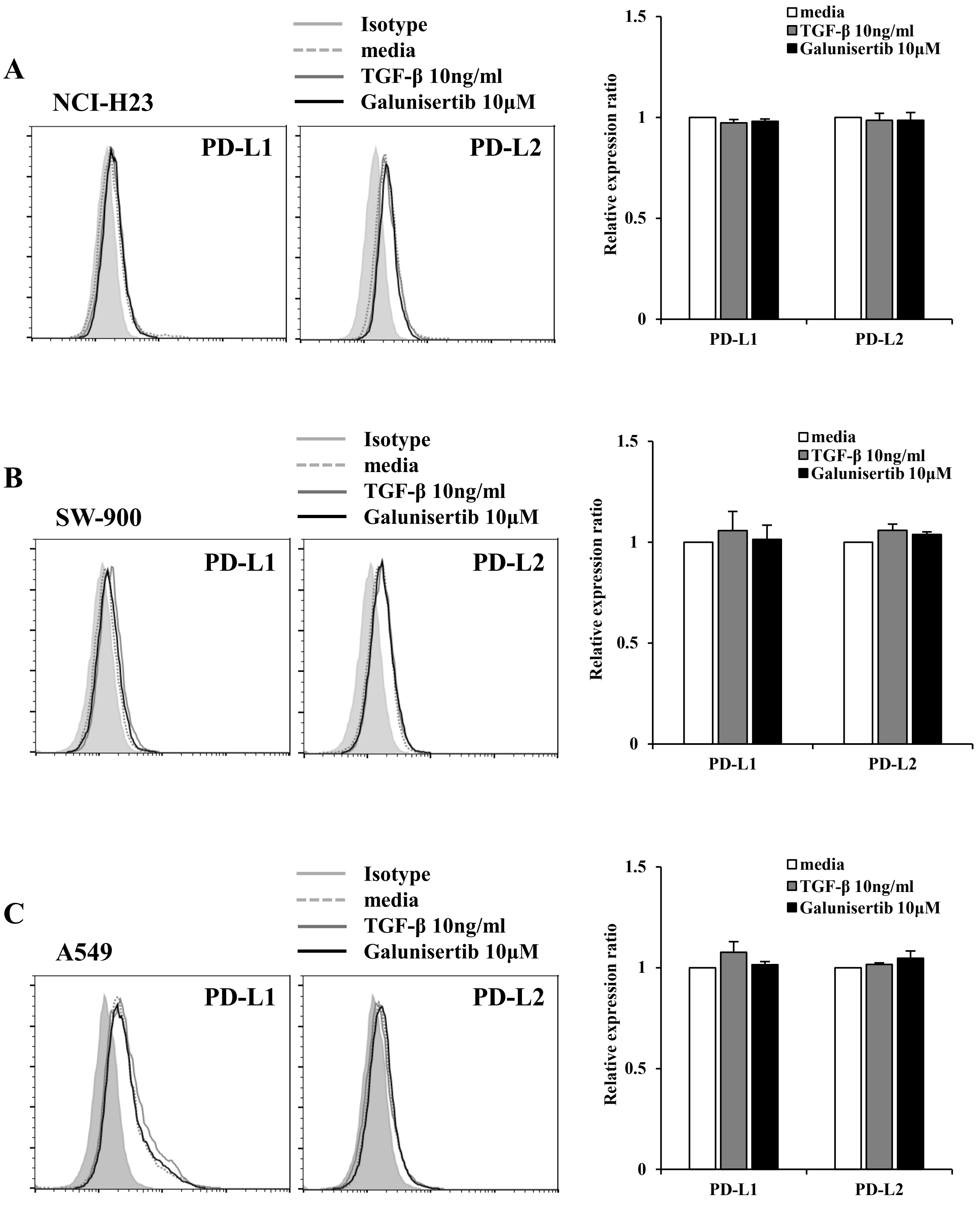

Supplementary Figure 1. The expression of PD-L1/2 on lung cancer cells; **(A)** NCI-H23, **(B)** SW-900, and **(C)** A549. Filled gray, dotted gray, gray and black lines represent isotype, media control, TGF-β 10 ng/ml and Galunisertib 10 μM treatment, respectively. The experiments were performed three times.
